# Academic outcomes before and after clinical onset of acquired demyelinating syndromes in children: a matched cohort data linkage study

**DOI:** 10.1002/acn3.52198

**Published:** 2024-10-02

**Authors:** Michael Eyre, Michael Absoud, Omar Abdel‐Mannan, Sarah Crichton, Yael Hacohen, Thomas Rossor, Sarah Rudebeck, Gavin Giovannoni, Ming Lim, Cheryl Hemingway

**Affiliations:** ^1^ Department of Biomedical Engineering School of Biomedical Engineering & Imaging Sciences, King's College London London UK; ^2^ Children's Neurosciences Evelina London Children's Hospital at Guy's and St Thomas' NHS Foundation Trust London UK; ^3^ Department of Women and Children's Health, Faculty of Life Sciences and Medicine School of Life Course Sciences, King's College London London UK; ^4^ Queen Square MS Centre, Faculty of Brain Sciences UCL Queen Square Institute of Neurology, University College London London UK; ^5^ Department of Neurology Great Ormond Street Hospital for Children London UK; ^6^ Department of Neuropsychology King's College Hospital London UK; ^7^ Faculty of Medicine and Dentistry Blizard Institute, Queen Mary University of London London UK

## Abstract

It is unknown if cognition is impaired before clinical onset of paediatric acquired demyelinating syndromes. We conducted a matched cohort study using prospectively collected educational data in multiple sclerosis (MS) and myelin oligodendrocyte glycoprotein antibody disease (MOGAD) patients (*n* = 60) and controls (pooled *n* = 449,553). Academic performance at ages 10–11 was impaired in MOGAD (−1.27 adjusted *z*‐score [95% CI: −1.81 to −0.73], *P* < 0.001) and preclinical MS (−0.40 [−0.80 to −0.0003], *P* = 0.0498). Moderate/high‐efficacy MS treatment was associated with better final academic performance (0.92 [0.28–1.57], *P* = 0.005). After clinical onset MS patients missed 8.7% of school (controls 2.9%, *P* < 0.001) and MOGAD patients 11.9% (controls 2.0%, *P* < 0.001).

## Introduction

Acquired demyelinating syndromes (ADS) such as multiple sclerosis (MS) and myelin oligodendrocyte glycoprotein antibody disease (MOGAD) often cause cognitive impairment and fatigue in children and adults.^1^, ^2^ Paediatric‐onset MS (POMS) is associated with worse cognitive impairment in adulthood compared to adult‐onset MS and reduced participation in university education and employment;^3^, ^4^ however, the impact of POMS on school participation is unknown.

Growing evidence supports the existence of a prodromal stage of MS, which may include preclinical cognitive problems.^5^, ^6^, ^7^ We used a prospectively collected national educational database to evaluate academic performance and school attendance before and after clinical onset of ADS in children. We hypothesised worse performance and attendance in MS and MOGAD compared to controls and better outcomes associated with moderate/high‐efficacy disease‐modifying therapy (MHE‐DMT) for MS.

## Methods

Patients were retrospectively identified from two paediatric neurology centres (London, UK). Eligible for inclusion were those meeting current diagnostic criteria for MS or MOGAD (including monophasic MOGAD),^8^, ^9^ aged <18 years at disease onset, who attended ≥1 UK state school. Controls were all pupils who attended a patient's school (1995–2021), linked via the Department for Education National Pupil Database (NPD). The study was approved by Wales Research Ethics Committee 6 (14/WA/0170). Written informed consent was obtained for each patient. The STROBE checklist for cohort studies was followed. Data on sex, ethnicity, Income Deprivation Affecting Children Index (IDACI) score, school attendance and attainment were extracted from NPD data sets (Table S1). Sociodemographic characteristics were compared between MS and MOGAD using chi‐squared, Fisher exact or Mann–Whitney *U*‐tests as appropriate. MHE‐DMTs were defined as treatments with efficacy of dimethyl fumarate or higher.^10^

Academic attainment was evaluated at the five key stages (KS1‐KS5, ages 5–18) of the national curriculum (NC), each of which ends with nationally standardised assessments or teacher‐assessed (TA) evaluations of academic progress (Table S2). Assessments analysed were KS1 TA points scores in reading, writing and maths (ages 6–7); KS2 standard assessment test (SAT) scores in reading, writing and maths (ages 10–11); KS3 TA NC levels in English, maths and science (ages 13–14); KS4 general certificate of secondary education and equivalents total points score (ages 15–16); and KS5 advanced level and equivalents total points score (ages 17–18). KS1 and KS3 assessments were compared between patients and controls (matched up to 10:1 on school, calendar year, sex, ethnicity and IDACI score) with the Mann–Whitney *U*‐test. KS2, KS4 and KS5 scores were converted to z‐scores within each calendar year then analysed with linear regression models. The first model evaluated the effect of group (preclinical MS, MS post‐onset or MOGAD post‐onset) on overall KS2 performance (mean SAT *z*‐score) adjusting for sex, ethnicity, school and IDACI quintile. A second model evaluated the effect of group (MS on MHE‐DMT, MS not on MHE‐DMT or MOGAD) on final academic performance (KS5 *z*‐score if available, otherwise KS4 *z*‐score) with the same covariates.

For school attendance we analysed each patient's termly time series relative to their first clinical event. For each term within 3 years of clinical onset, and the first three full years after onset, absence was compared between patients and controls (matched up to 10:1 on school, school year, calendar year, sex, ethnicity and IDACI score) using the Mann–Whitney *U*‐test, with false discovery rate corrected *P*‐values for the termly comparisons. Estimated days missed due to ADS per year were calculated from the differences between the patient and control medians. Attendance 2 years before and after establishment on MHE‐DMT was compared using the Mann–Whitney *U*‐test. Analyses are further detailed in Data S1.

## Results

Sixty patients were recruited: 38 MS (30 female, median age at onset 14.2 years [IQR: 11.7–15.3]) and 22 MOGAD (13 female, median 5.8 years [IQR: 3.2–7.4], 11 relapsing) **(**Table 1). MS patients lived in areas with median 27.6% of children affected by income deprivation (IQR: 18.5–41.6%) versus 9.2% (IQR: 6.4–29.2%) for MOGAD (*p* = .006). The total pool of linked controls comprised 449,553 children.

**Table 1:** Patient characteristics.

Variable	MS (*n* = 38)	MOGAD (*n* = 22)	*P*
Age at clinical onset (years), median (IQR)	14.2 (11.7–15.3)	5.8 (3.2–7.4)	–
Female, *n* (%)	30 (79%)	13 (59%)	0.1
Ethnic group^a^			
White, *n*/*N* (%)	13/37 (35%)	15/22 (68%)	0.01
Asian, *n*/*N* (%)	8/37 (22%)	–^b^	>0.05^b^
Black, *n*/*N* (%)	10/37 (27%)	–^b^	<0.05^b^
Other, *n*/*N* (%)	6/37 (16%)	–^b^	>0.05^b^
Income deprivation Affecting Children Index score, median (IQR)	0.276 (0.185–0.416)	0.092 (0.064–0.292)	0.006
Number of clinical events, median (IQR)	2 (2–4)	1.5 (1–7)	–
MRI brain lesions, no. patients (%)	38 (100%)	18 (82%)	–
MHE‐DMT for MS, no. patients (%)	24 (63%)^c^	–	–
Follow‐up duration (years), median (IQR)	4.7 (3.7–5.9)	7.3 (3.4–9.7)	–

CNS, central nervous system; MHE‐DMT, moderate/high‐efficacy disease‐modifying therapy; MS, multiple sclerosis; MOGAD, myelin oligodendrocyte glycoprotein antibody disease.

^a^ Ethnic group was not recorded in one MS patient.

^b^ Exact values not provided due to statistical disclosure risk. Black ethnicity was significantly more frequent in MS compared to MOGAD.

^c^ MHE‐DMTs for MS were fingolimod (7), natalizumab (7), dimethyl fumarate (6) and ocrelizumab (4).

At ages 6–7 (KS1) the preclinical MS group (*n* = 33) had no significant differences in attainment from controls; the MOGAD group (four preclinical and seven post‐onset) performed worse than controls in writing (median TA points score 13 [IQR: 11–16] vs. 15 [13–17], *P* = 0.049) and maths (15 [13–17] vs. 17 [13–21], *P* = 0.04) (Table S3).

**Figure 1:**
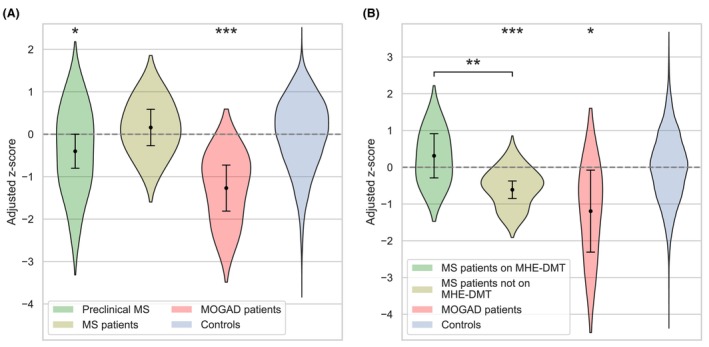
Academic performance in nationally standardised assessments. Academic performance in the key stage 2 SAT at ages 10–11 (A) and at the final academic assessment (Key Stage 4/5) (B). At key stage 2 the MS group are separated according to the timing of the SAT relative to their first clinical event, while the MOGAD group were all post‐onset; at the final academic assessment all patients were post‐onset. Violin plots show the residualised z‐scores after adjustment for sex, ethnicity, school and IDACI quintile. Overlaid in black are the estimated group effects (relative to controls) and 95% CIs in the full linear models with statistical significance indicated by asterisks (*P < 0.05, **P < 0.01, ***P < 0.001). IDACI, Income Deprivation Affecting Children Index; MHE‐DMT, moderate/high‐efficacy disease‐modifying therapy; MS, multiple sclerosis; MOGAD, myelin oligodendrocyte glycoprotein antibody disease; SAT, standard assessment test.

At ages 10–11 (KS2) the preclinical MS group (*n* = 24) performed worse than controls (*n* = 10,520) with a −0.40 adjusted *z*‐score difference (95% CI: −0.80 to −0.0003, *P* = 0.0498), MS patients (*n* = 7) had no significant difference from controls and MOGAD patients (*n* = 7) performed worse than controls (−1.27 [−1.81 to −0.73], *P* < 0.001) (Fig. 1A, Table S4).

At ages 13–14 (KS3) the MS group (three preclinical and four post‐onset) had no significant differences from controls; MOGAD patients (*n* = 3) performed worse than controls in English (median TA NC level 4 vs. 6 [IQR: 6–5], *P* = 0.04) and maths (3 vs. 6 [5–7], *P* = 0.01) (Table S3).

At final academic assessment (KS4/5) MS patients on MHE‐DMT (*n* = 6) had no significant difference from controls (*n* = 12,847), less‐treated MS patients (*n* = 15) performed worse than both controls (−0.61 *z*‐score difference [95% CI: −0.85 to −0.38], *P* < 0.001) and those on MHE‐DMT (−0.92 [−1.57 to −0.28], *P* = 0.005), and MOGAD patients (*n* = 3) performed worse than controls (−1.19 [−2.31 to −0.08], *P* = 0.036) (Fig. 1B, Tables S5 and S6).

**Figure 2:**
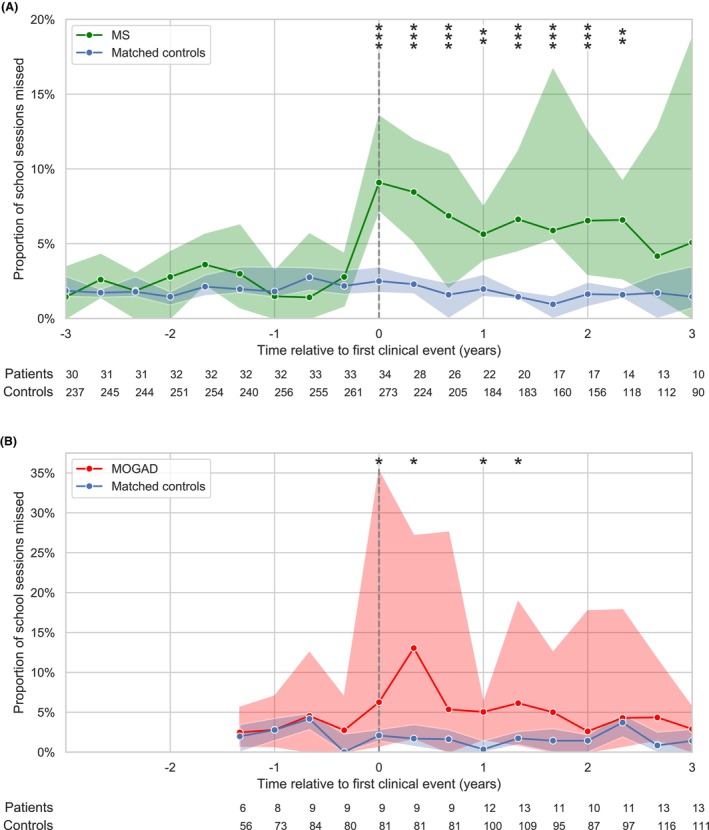
School absence before and after clinical onset of MS and MOGAD. The median proportion of sessions absent per school term in MS (A: green) and MOGAD (B: red) compared to matched controls (blue). Patients' time series were centred on the term when their first clinical event occurred (vertical dashed line). Shaded regions indicate the 95% CI for the group median at each timepoint. The group sizes at each timepoint are shown below; timepoints with data available in ≤5 patients were not analysed. Asterisks indicate statistical significance (false discovery rate corrected p‐values) at each timepoint (*P < 0.05, **P < 0.01 and ***P < 0.001). MS, multiple sclerosis; MOGAD, myelin oligodendrocyte glycoprotein antibody disease.

There were no significant differences in school absence prior to clinical onset (Fig. 2). In the first post‐onset year MS patients (*n* = 33) missed a median 8.7% (IQR: 6.5–13.4%) of school (controls [*n* = 264] 2.9% [1.2–5.8%], *P* < 0.001); 49% missed ≥10%. Estimated days missed due to MS were 11.0 in the first year (95% CI: 7.6–18.0), 6.3 (5.1–16.5) in the second and 5.9 (2.4–13.0) in the third. Median absence before and after MHE‐DMT for MS was 11.9% (IQR: 6.5–16.0%, *n* = 18) and 6.5% (4.2–9.4%, *n* = 8), respectively (*P* = 0.08). MOGAD patients (*n* = 9) missed a median 11.9% (IQR: 6.5–24.4%) of school in the first year (controls [*n* = 81] 2.0% [1.3–5.4%], *P* < 0.001); 57% missed ≥10%. Estimated days missed due to MOGAD were 17.6 in the first year (95% CI: −0.5 to 42.4), 6.1 (1.2–14.5) in the second and 4.9 (−0.7 to 30.2) in the third.

## Discussion

Our main findings were worse academic performance in MOGAD, preclinical MS and less‐treated MS and increased school absence after clinical onset of MS and MOGAD.

Children who later developed MS scored lower than controls on standardised tests at ages 10–11 (mean 34th centile after sociodemographic adjustment), a novel finding in POMS and together with previous findings in adult‐onset MS suggesting a possible preclinical cognitive endophenotype.^5^, ^6^ It remains unclear to what extent this indicates prodromal neuroinflammation as opposed to an MS susceptibility state,^7^ perhaps correlated with academic performance due to genetic or environmental factors. MS patients with clinical onset before ages 10–11 trended towards better performance at this timepoint compared to the preclinical group; this may reflect treatment benefit, but should be interpreted with caution due to small numbers.

School absence after clinical onset was threefold higher in MS and nearly sixfold higher in MOGAD compared to controls. High rates of school discontinuation were previously reported in POMS.^11^ Children may be absent due to physical limitations, healthcare appointments, mental health and notably fatigue,^12^ which is strongly associated with work absence in adult‐onset MS.^13^

MOGAD patients had the worst academic performance post‐onset (mean 10th centile at ages 10–11). This cannot be solely attributed to school absence, which trended towards normal within 2 years post‐onset (Fig. 2B); cognitive deficits likely contribute, especially following early/recurrent acute disseminated encephalomyelitis,^14^, ^15^ consistent with observed deviations in expected brain growth.^16^ MS patients on no or less efficacious DMTs performed worse than controls at the final academic assessment (mean 27th centile) and nearly one standard deviation below those receiving MHE‐DMT. Escalation to high‐efficacy DMT for MS is associated not only with reduced relapse rate but also reduced brain atrophy and potentially protection from cognitive decline.^17^, ^18^

This study was mainly limited by a small, retrospective cohort. There is a high risk of severity bias in the MOGAD group, as prevalent patients under follow‐up when antibody testing first became available likely had a relapsing course, which may correlate with worse cognitive impairments. These older MOGAD patients disproportionately influence the academic performance data in later key stages, as more recently diagnosed patients have not yet completed their education. Consequently these data may not be representative of incident MOGAD patients today, who furthermore may benefit from evolving immunotherapeutic strategies.^19^ There may also be bias in patients selected for MHE‐DMT. Nevertheless, our findings highlight significant educational disruptions and academic challenges posed by ADS, emphasising the importance of monitoring and support for affected patients^20^ and adding to growing evidence of preclinical cognitive differences in MS.

## Author Contributions

Conception and design of the study: M.E., G.G., M.L. and C.H. Acquisition and analysis of data: M.E., M.A., O.A‐M., S.C., Y.H., T.R., M.L. and C.H. Drafting of the manuscript and figures: M.E., M.A., S.R., G.G., M.L. and C.H.

## Conflict of Interest

None declared.

## Supporting information


**Data S1.** Supplementary methods.

**Table S1.** National pupil database datasets.

**Table S2.** Overview of UK national curriculum key stages.

**Table S3.** Academic performance in teacher‐assessed evaluations of academic progress.

**Table S4.** Linear model for Key Stage 2 Standard Assessment Test performance.

**Table S5.** Linear model for final academic performance (reference group: Controls).

**Table S6.** Linear model for final academic performance (reference group: MS patients not on MHE‐DMT).

## Data Availability

Anonymised data that support the findings of this study are available on request from the corresponding author. The data are not publicly available due to privacy restrictions.
