# Supplementary material for: Academic outcomes before and after clinical onset of acquired demyelinating syndromes in children: a matched cohort data linkage study
Source: Ann Clin Transl Neurol. 2024 Oct 2;11(11):3025–30. doi: 10.1002/acn3.52198 (PMC11572733; doi:10.1002/acn3.52198)
Supplement: Supplementary file 1 — Data S1. Supplementary methods. [file ACN3-11-3025-s006.docx]

# Supplementary Methods

***Clinical Data***

Patients were retrospectively identified from the clinic databases of Great Ormond Street Hospital for Children and Evelina London Children’s Hospital. Patients eligible for inclusion were contacted by their clinical team via telephone or email. Data retrieved from hospital electronic health records included date of birth, date of the first clinical event, number of relapses and date of commencing a moderate/high-efficacy disease-modifying therapy (MHE-DMT) for MS, defined as DMTs with efficacy classification of dimethyl fumarate or higher (alemtuzumab, ofatumumab, ocrelizumab, natalizumab, cladribine, fingolimod or ozanimod [1.0mg]).[^1^](https://sciwheel.com/work/citation?ids=15988447&pre=&suf=&sa=0&dbf=0)

***Sociodemographic Data***

Sociodemographic data were extracted from the National Pupil Database (NPD) Spring Census datasets (**Supplementary Table S1**). The Income Deprivation Affecting Children Index (IDACI) score is derived from the home postcode of each pupil and indicates the proportion of children in the pupil’s local area living in income-deprived families. The IDACI rank indicates the position of the local area nationally. IDACI ranks were converted to quintiles, in which participants in the first quintile lived in the top 20% most deprived areas. IDACI scores and quintiles were converted to annual time series for each participant. The mean IDACI score over time was used for the summary of patient characteristics.

***Academic Performance Data***

The NPD Key Stage 1 to 5 datasets used are detailed in **Supplementary Table S1**. The maximum number of matched controls per patient for Key Stage 1 and Key Stage 3 assessments was set at 10; if more than 10 matches were available those with the closest IDACI score to the patient were selected, with missing IDACI scores imputed from the closest available score in that participant’s time series (e.g. the previous or following year’s score). For the Key Stage 2 linear model MS and MOGAD patients were separated into those taking the SAT before versus after their first clinical event (excluding groups smaller than three). For the final academic assessment (Key Stage 4/5) linear model MS patients were separated into those established on MHE-DMT at the time of assessment versus those not; patients with their first clinical event after the final assessment were not included. The date of all assessments was pragmatically assumed to be 15 May (the typical start date for summer examinations in the UK). Establishment on MHE-DMT was pragmatically defined as having occurred three months (90 days) after administration of the first dose. Controls included in the linear models were all participants who took the assessment at the same school as an included patient (contemporaneously or not). Missing IDACI quintiles were imputed from within each participant’s time series as described above. Models were fit with heteroscedasticity-consistent covariance matrix estimation to compute robust standard errors. Estimates of group effects (coefficients) and corresponding 95% CIs and p-values were extracted from the models with ‘Controls’ as the reference group (**Supplementary Tables S4-S5**); the final academic assessment model was also run with ‘MS patients not on MHE-DMT’ as the reference group (**Supplementary Table S6**) in order to derive a 95% CI and p-value for the difference between the two MS groups.

***School Absence Data***

The NPD absence datasets (**Supplementary Table S1**) were used to determine the total number of school sessions possible and missed (due to authorised or unauthorised absence) for each participant over time, converted to a termly time series. Absence proportion was calculated as the number of sessions missed divided by the total possible. Patients’ attendance time series were centred on the term when the symptoms of their first clinical event started; events starting during school holidays carried over to the following term. The maximum number of matched controls per patient at each timepoint was set at 10; if more than 10 matches were available those with the closest IDACI score to the patient were selected. Missing IDACI scores were imputed from within each participant’s time series as described above. Participants with <10 sessions possible were not included at that timepoint. Timepoints with data available in ≤5 patients were not analysed. The estimated number of school days missed due to MS or MOGAD per year was the difference between the patient and control group median absence proportions multiplied by 190 (the average number of days in a UK school year). 95% CIs for termly group medians (**Fig 2**) and differences between group medians (for calculation of estimated school days missed) were calculated from the bootstrap distributions obtained by resampling with replacement over 10,000 folds. School absence was also compared in MS patients before and after establishment on MHE-DMT, calculated across all terms within two years before (excluding terms prior to the first clinical disease event) or after establishment on MHE-DMT.

***Statistical Analysis Tools***

Analyses were conducted using Python 3.7 with scipy, statsmodels and sklearn.

[1.  Samjoo, I.A., Worthington, E., Drudge, C., Zhao, M., Cameron, C., Häring, D.A., Stoneman, D., Klotz, L., and Adlard, N. (2021). Efficacy classification of modern therapies in multiple sclerosis. J. Comp. Eff. Res. *10*, 495–507. 10.2217/cer-2020-0267.](https://sciwheel.com/work/bibliography/15988447)
