# Supplementary material for: Academic outcomes before and after clinical onset of acquired demyelinating syndromes in children: a matched cohort data linkage study
Source: Ann Clin Transl Neurol. 2024 Oct 2;11(11):3025–30. doi: 10.1002/acn3.52198 (PMC11572733; doi:10.1002/acn3.52198)
Supplement: Supplementary file 2 — Table S1. National pupil database datasets. [file ACN3-11-3025-s002.docx]

# Supplementary Table S1: National Pupil Database Datasets

| **Dataset description** | **No. records** |
| --- | --- |
| Key Stage 1 Pupil data for academic years (AY) 1997/1998 to 2018/2019 for patients plus a group of matched controls with the same school unique reference number (URN) | 64,532 |
| Key Stage 2 Pupil data for AYs 1995/1996 to 2018/2019 for patients plus controls with the same school URN | 61,400 |
| Key Stage 3 Teacher Assessment data for AYs 2008/2009 to 2012/2013 for patients plus controls with the same school URN | 7,709 |
| Key Stage 4 Pupil data for AYs 2001/2002 to 2013/2014 for patients plus controls with the same school URN | 10,770 |
| Key Stage 4 Pupil data for AYs 2014/2015 to 2018/2019 for patients plus controls with the same school URN | 12,789 |
| Key Stage 5 Student data for AYs 2001/2002 to 2018/2019 (except for 2015/2016 - see below) for patients plus controls with the same school URN | 433,284 |
| Key Stage 5 Student data for AY 2015/2016 for patients plus controls with the same school URN | 18,433 |
| Key Stage 5 Exam data for AY 2015/2016 for pupils in the dataset above (used to link with URN) | 76,672 |
| 3 Term Absence data for AY 2006/2007 for patients plus controls with the same school URN | 4,159 |
| 3 Term Absence data for AY 2007/2008 for patients plus controls with the same school URN | 7,165 |
| 3 Term Absence data for AY 2008/2009 for patients plus controls with the same school URN | 11,385 |
| 3 Term Absence data for AY 2009/2010 for patients plus controls with the same school URN | 14,870 |
| 3 Term Absence data for AY 2010/2011 for patients plus controls with the same school URN | 18,172 |
| 3 Term Absence data for AY 2011/2012 for patients plus controls with the same school URN | 19,075 |
| 3 Term Absence data for AY 2012/2013 for patients plus controls with the same school URN | 20,665 |
| 3 Term Absence data for AY 2013/2014 for patients plus controls with the same school URN | 20,798 |
| 3 Term Absence data for AY 2014/2015 for patients plus controls with the same school URN | 24,042 |
| 3 Term Absence data for AY 2015/2016 for patients plus controls with the same school URN | 30,278 |
| 3 Term Absence data for AY 2016/2017 for patients plus controls with the same school URN | 31,840 |
| 3 Term Absence data for AY 2017/2018 for patients plus controls with the same school URN | 31,473 |
| 3 Term Absence data for AY 2018/2019 for patients plus controls with the same school URN | 28,786 |
| 2 Term Absence data for AY 2020/2021 for patients plus controls with the same school URN | 18,243 |
| Spring Census data for AY 2006/2007 for all ‘on roll’ patients plus controls with the same school URN | 9,303 |
| Spring Census data for AY 2007/2008 for all ‘on roll’ patients plus controls with the same school URN | 13,663 |
| Spring Census data for AY 2008/2009 for all ‘on roll’ patients plus controls with the same school URN | 16,366 |
| Spring Census data for AY 2009/2010 for all ‘on roll’ patients plus controls with the same school URN | 18,545 |
| Spring Census data for AY 2010/2011 for all ‘on roll’ patients plus controls with the same school URN | 22,017 |
| Spring Census data for AY 2011/2012 for all ‘on roll’ patients plus controls with the same school URN | 23,326 |
| Spring Census data for AY 2012/2013 for all ‘on roll’ patients plus controls with the same school URN | 25,142 |
| Spring Census data for AY 2013/2014 for all ‘on roll’ patients plus controls with the same school URN | 29,353 |
| Spring Census data for AY 2014/2015 for all ‘on roll’ patients plus controls with the same school URN | 30,610 |
| Spring Census data for AY 2015/2016 for all ‘on roll’ patients plus controls with the same school URN | 34,976 |
| Spring Census data for AY 2016/2017 for all ‘on roll’ patients plus controls with the same school URN | 37,451 |
| Spring Census data for AY 2017/2018 for all ‘on roll’ patients plus controls with the same school URN | 36,974 |
| Spring Census data for AY 2018/2019 for all ‘on roll’ patients plus controls with the same school URN | 37,066 |
| Spring Census data for AY 2019/2020 for all ‘on roll’ patients plus controls with the same school URN | 37,709 |
| Spring Census data for AY 2020/2021 for all ‘on roll’ patients plus controls with the same school URN | 27,882 |

AY, academic year; URN, unique reference number.
