# Supplementary material for: Academic outcomes before and after clinical onset of acquired demyelinating syndromes in children: a matched cohort data linkage study
Source: Ann Clin Transl Neurol. 2024 Oct 2;11(11):3025–30. doi: 10.1002/acn3.52198 (PMC11572733; doi:10.1002/acn3.52198)
Supplement: Supplementary file 3 — Table S2. Overview of UK national curriculum key stages. [file ACN3-11-3025-s007.docx]

# Supplementary Table S2: Overview of UK National Curriculum Key Stages

| **Key Stage** | **National Curriculum years** | **Typical age at end of Key Stage** | **Assessment type at end of Key Stage** |
| --- | --- | --- | --- |
| 1 | 1-2 | 6-7 years | Teacher-assessed National Curriculum level |
| 2 | 3-6 | 10-11 years | Standard Assessment Tests (SATs) |
| 3 | 7-9 | 13-14 years | Teacher-assessed National Curriculum level |
| 4 | 10-11 | 15-16 years | General Certificate of Secondary Education (GCSE) and equivalents |
| 5 | 12-13 | 17-18 years | Advanced Level (A-Level) and equivalents |
