# Supplementary material for: Academic outcomes before and after clinical onset of acquired demyelinating syndromes in children: a matched cohort data linkage study
Source: Ann Clin Transl Neurol. 2024 Oct 2;11(11):3025–30. doi: 10.1002/acn3.52198 (PMC11572733; doi:10.1002/acn3.52198)
Supplement: Supplementary file 4 — Table S3. Academic performance in teacher‐assessed evaluations of academic progress. [file ACN3-11-3025-s001.docx]

# Supplementary Table S3: Academic Performance in Teacher-Assessed Evaluations of Academic Progress

|  | **MS** | | **MS matched controls** | |  | **MOGAD** | | **MOGAD matched controls** | |  |
| --- | --- | --- | --- | --- | --- | --- | --- | --- | --- | --- |
| **Assessment** | **N** | **Score, median (IQR)** | **N** | **Score, median (IQR)** | ***p*** | **N** | **Score, median (IQR)** | **N** | **Score, median (IQR)** | ***p*** |
| Key Stage 1 TA points score (ages 6-7)^a^ |  |  |  |  |  |  |  |  |  |  |
| Reading | 33 | 15 (13-17) | 245 | 15 (13-17) | 0.5 | 11^b^ | 17 (14-17) | 96 | 17 (15-21) | 0.2 |
| Writing | 33 | 15 (13-17) | 245 | 15 (13-17) | 0.3 | 11^b^ | 13 (11-16) | 96 | 15 (13-17) | 0.049 |
| Maths | 33 | 15 (13-17) | 245 | 15 (13-17) | 0.5 | 11^b^ | 15 (13-17) | 96 | 17 (13-21) | 0.04 |
| Key Stage 3 TA NC level (ages 13-14)^c^ |  |  |  |  |  |  |  |  |  |  |
| English | 7^d^ | 6 (4.5-7) | 60 | 6 (5-7) | 0.5 | 3 | 4^e^ | 15 | 6 (5-6) | 0.04 |
| Maths | 7^d^ | 6 (6-7.5) | 60 | 6 (5-7) | 0.3 | 3 | 3^e^ | 17 | 6 (5-7) | 0.01 |
| Science | 7^d^ | 6 (5-7) | 60 | 6 (5-7) | 0.5 | 3 | 4^e^ | 19 | 5 (4.5-6) | 0.08 |

a. A teacher-assessed points score <13 at this age indicates a child working below the expected level, 13 = lower end of the expected level, 15 = comfortably at the expected level, 17 = top end of the expected level, >17 = above the expected level.

b. Among the MOGAD group at Key Stage 1, seven had their first clinical event prior to the assessment (post-onset), and four after (preclinical). The scores for these subgroups are provided below:

|  | **Post-onset MOGAD** | | **Matched controls** | |  | **Preclinical MOGAD** | | **Matched controls** | |  |
| --- | --- | --- | --- | --- | --- | --- | --- | --- | --- | --- |
| **Assessment** | **N** | **Score, median (IQR)** | **N** | **Score, median (IQR)** | ***p*** | **N** | **Score, median (IQR)** | **N** | **Score, median (IQR)** | ***p*** |
| Key Stage 1 TA points score (ages 6-7)^a^ |  |  |  |  |  |  |  |  |  |  |
| Reading | 7 | 15 (14-17) | 56 | 17 (15-21) | 0.1 | 4 | 17^e^ | 40 | 17 (14.5-21) | 0.4 |
| Writing | 7 | 13 (11-16) | 56 | 15 (15-17) | 0.06 | 4 | 14^e^ | 40 | 15 (13-17) | 0.3 |
| Maths | 7 | 13 (13-15) | 56 | 15 (13-21) | 0.03 | 4 | 17^e^ | 40 | 17 (14.5-21) | 0.3 |

c. A teacher-assessed National Curriculum level <5 at this age indicates a child working below the expected level (level 4 should be achieved at ages 10-11), 5 = lower end of the expected level, 6 = comfortably at the expected level, 7 = top end of the expected level, >7 = above the expected level.

d. Among the MS group at Key Stage 3, four had their first clinical event prior to the assessment (post-onset), and three after (preclinical). The scores for these subgroups are provided below:

|  | **Post-onset MS** | | **Matched controls** | |  | **Preclinical MS** | | **Matched controls** | |  |
| --- | --- | --- | --- | --- | --- | --- | --- | --- | --- | --- |
| **Assessment** | **N** | **Score, median (IQR)** | **N** | **Score, median (IQR)** | ***p*** | **N** | **Score, median (IQR)** | **N** | **Score, median (IQR)** | ***p*** |
| Key Stage 3 TA NC level (ages 13-14)^c^ |  |  |  |  |  |  |  |  |  |  |
| English | 4 | 5.5^e^ | 30 | 6 (5-7) | 0.4 | 3 | 6^e^ | 30 | 6 (5-6) | 0.4 |
| Maths | 4 | 7^e^ | 30 | 7 (6-7) | 0.4 | 3 | 6^e^ | 30 | 6 (5-7) | 0.2 |
| Science | 4 | 6^e^ | 30 | 6 (5.3-7) | 0.5 | 3 | 6^e^ | 30 | 6 (5-7) | 0.4 |

e. IQR not provided due to statistical disclosure risk.

MS, multiple sclerosis; MOGAD, myelin oligodendrocyte glycoprotein antibody disease; NC, National Curriculum; TA, teacher-assessed.
