# Supplementary material for: Academic outcomes before and after clinical onset of acquired demyelinating syndromes in children: a matched cohort data linkage study
Source: Ann Clin Transl Neurol. 2024 Oct 2;11(11):3025–30. doi: 10.1002/acn3.52198 (PMC11572733; doi:10.1002/acn3.52198)
Supplement: Supplementary file 5 — Table S4. Linear model for Key Stage 2 Standard Assessment Test performance. [file ACN3-11-3025-s003.docx]

# Supplementary Table S4: Linear model for Key Stage 2 Standard Assessment Test performance

Generalized Linear Model Regression Results

==============================================================================

Dep. Variable: Score No. Observations: 10558

Model: GLM Df Residuals: 10506

Model Family: Gaussian Df Model: 51

Link Function: identity Scale: 0.72234

Method: IRLS Log-Likelihood: -13238.

Date: Thu, 25 May 2023 Deviance: 7588.9

Time: 13:14:01 Pearson chi2: 7.59e+03

No. Iterations: 3

Covariance Type: HC1

=====================================================================================================================

coef std err z P>|z| [0.025 0.975]

---------------------------------------------------------------------------------------------------------------------

Intercept 0.7101 0.083 8.565 0.000 0.548 0.873

Diagnosis[T.MOGAD patients] -1.2696 0.277 -4.586 0.000 -1.812 -0.727

Diagnosis[T.MS patients] 0.1586 0.219 0.726 0.468 -0.270 0.587

Diagnosis[T.Preclinical MS] -0.4013 0.205 -1.961 0.0498 -0.802 -0.000

C(Ethnic_group)[T.ASIA] 0.1748 0.046 3.780 0.000 0.084 0.265

C(Ethnic_group)[T.BLAC] -0.0005 0.045 -0.011 0.991 -0.090 0.089

C(Ethnic_group)[T.CHIN] 0.4265 0.098 4.353 0.000 0.234 0.619

C(Ethnic_group)[T.MIXD] 0.1153 0.052 2.213 0.027 0.013 0.217

C(Ethnic_group)[T.UNCL] 0.1081 0.093 1.163 0.245 -0.074 0.290

C(Ethnic_group)[T.WHIT] 0.0429 0.043 0.993 0.321 -0.042 0.128

C(Gender)[T.M] -0.1271 0.017 -7.591 0.000 -0.160 -0.094

C(School)[1] -0.5556 0.080 -6.916 0.000 -0.713 -0.398

C(School)[2] -0.9251 0.115 -8.013 0.000 -1.151 -0.699

C(School)[3] -0.0342 0.073 -0.466 0.641 -0.178 0.110

C(School)[4] -0.6392 0.081 -7.847 0.000 -0.799 -0.480

C(School)[5] -0.4298 0.077 -5.568 0.000 -0.581 -0.278

C(School)[6] -0.7181 0.079 -9.120 0.000 -0.872 -0.564

C(School)[7] -0.0017 0.074 -0.023 0.981 -0.147 0.144

C(School)[8] -0.7261 0.100 -7.234 0.000 -0.923 -0.529

C(School)[9] -0.8251 0.090 -9.139 0.000 -1.002 -0.648

C(School)[10] -0.2317 0.072 -3.234 0.001 -0.372 -0.091

C(School)[11] 0.0581 0.075 0.773 0.440 -0.089 0.205

C(School)[12] -0.7657 0.077 -9.898 0.000 -0.917 -0.614

C(School)[13] -0.7531 0.083 -9.026 0.000 -0.917 -0.590

C(School)[14] -0.7071 0.076 -9.261 0.000 -0.857 -0.557

C(School)[15] -0.5894 0.070 -8.457 0.000 -0.726 -0.453

C(School)[16] -0.7051 0.080 -8.855 0.000 -0.861 -0.549

C(School)[17] -0.2684 0.095 -2.816 0.005 -0.455 -0.082

C(School)[18] -0.4551 0.081 -5.611 0.000 -0.614 -0.296

C(School)[19] -0.3739 0.077 -4.886 0.000 -0.524 -0.224

C(School)[20] -0.8013 0.090 -8.903 0.000 -0.978 -0.625

C(School)[21] -0.7624 0.088 -8.676 0.000 -0.935 -0.590

C(School)[22] -0.6014 0.081 -7.416 0.000 -0.760 -0.442

C(School)[23] -0.9269 0.095 -9.802 0.000 -1.112 -0.742

C(School)[24] -0.2608 0.181 -1.438 0.150 -0.616 0.095

C(School)[25] -0.6991 0.136 -5.144 0.000 -0.966 -0.433

C(School)[26] -0.1896 0.087 -2.176 0.030 -0.360 -0.019

C(School)[27] -0.4893 0.082 -5.952 0.000 -0.650 -0.328

C(School)[28] -0.3303 0.086 -3.836 0.000 -0.499 -0.162

C(School)[29] -0.0290 0.073 -0.394 0.693 -0.173 0.115

C(School)[30] 0.0143 0.084 0.171 0.864 -0.150 0.178

C(School)[31] -0.4503 0.085 -5.306 0.000 -0.617 -0.284

C(School)[32] -0.7703 0.083 -9.268 0.000 -0.933 -0.607

C(School)[33] -0.2214 0.080 -2.766 0.006 -0.378 -0.064

C(School)[34] 0.4354 0.213 2.046 0.041 0.018 0.852

C(School)[35] -0.2481 0.088 -2.822 0.005 -0.420 -0.076

C(School)[36] -0.4447 0.102 -4.372 0.000 -0.644 -0.245

C(School)[37] -0.4929 0.090 -5.455 0.000 -0.670 -0.316

C(IDACIQuintile, Treatment(reference="Q5"))[T.Q1] -0.3560 0.044 -8.077 0.000 -0.442 -0.270

C(IDACIQuintile, Treatment(reference="Q5"))[T.Q2] -0.2871 0.040 -7.156 0.000 -0.366 -0.208

C(IDACIQuintile, Treatment(reference="Q5"))[T.Q3] -0.2030 0.039 -5.158 0.000 -0.280 -0.126

C(IDACIQuintile, Treatment(reference="Q5"))[T.Q4] -0.0589 0.037 -1.601 0.109 -0.131 0.013

=====================================================================================================================
