# Supplementary material for: Academic outcomes before and after clinical onset of acquired demyelinating syndromes in children: a matched cohort data linkage study
Source: Ann Clin Transl Neurol. 2024 Oct 2;11(11):3025–30. doi: 10.1002/acn3.52198 (PMC11572733; doi:10.1002/acn3.52198)
Supplement: Supplementary file 7 — Table S6. Linear model for final academic performance (reference group: MS patients not on MHE‐DMT). [file ACN3-11-3025-s005.docx]

# Supplementary Table S6: Linear model for final academic performance (reference group: MS patients not on MHE-DMT)

Generalized Linear Model Regression Results

==============================================================================

Dep. Variable: Score No. Observations: 12871

Model: GLM Df Residuals: 12833

Model Family: Gaussian Df Model: 37

Link Function: identity Scale: 0.85629

Method: IRLS Log-Likelihood: -17246.

Date: Thu, 25 May 2023 Deviance: 10989.

Time: 11:42:59 Pearson chi2: 1.10e+04

No. Iterations: 3

Covariance Type: HC1

=====================================================================================================================

coef std err z P>|z| [0.025 0.975]

---------------------------------------------------------------------------------------------------------------------

Intercept -0.1800 0.134 -1.346 0.178 -0.442 0.082

Diagnosis[T.Controls] 0.6117 0.122 5.033 0.000 0.373 0.850

Diagnosis[T.MOGAD patients] -0.5824 0.582 -1.001 0.317 -1.722 0.558

Diagnosis[T.MS patients on MHE-DMT] 0.9233 0.330 2.796 0.005 0.276 1.570

C(Ethnic_group)[T.ASIA] 0.0418 0.045 0.939 0.348 -0.045 0.129

C(Ethnic_group)[T.BLAC] -0.1653 0.042 -3.893 0.000 -0.248 -0.082

C(Ethnic_group)[T.CHIN] 0.6152 0.114 5.376 0.000 0.391 0.840

C(Ethnic_group)[T.MIXD] -0.1799 0.050 -3.576 0.000 -0.278 -0.081

C(Ethnic_group)[T.UNCL] -0.4062 0.086 -4.728 0.000 -0.575 -0.238

C(Ethnic_group)[T.WHIT] -0.3129 0.042 -7.500 0.000 -0.395 -0.231

C(Gender)[T.M] -0.2592 0.018 -14.311 0.000 -0.295 -0.224

C(School)[1] 0.0491 0.045 1.097 0.273 -0.039 0.137

C(School)[2] 1.8479 0.046 39.824 0.000 1.757 1.939

C(School)[3] 0.6257 0.047 13.384 0.000 0.534 0.717

C(School)[4] 0.1709 0.068 2.514 0.012 0.038 0.304

C(School)[5] 0.6294 0.066 9.601 0.000 0.501 0.758

C(School)[6] 0.1568 0.050 3.114 0.002 0.058 0.256

C(School)[7] -1.9170 0.088 -21.665 0.000 -2.090 -1.744

C(School)[8] -0.4265 0.166 -2.565 0.010 -0.752 -0.101

C(School)[9] -0.1893 0.308 -0.615 0.538 -0.792 0.414

C(School)[10] 1.9251 0.129 14.978 0.000 1.673 2.177

C(School)[11] -2.5996 0.053 -48.682 0.000 -2.704 -2.495

C(School)[12] 0.3261 0.050 6.496 0.000 0.228 0.424

C(School)[13] -0.3218 0.053 -6.109 0.000 -0.425 -0.219

C(School)[14] 0.1731 0.044 3.895 0.000 0.086 0.260

C(School)[15] 0.3383 0.049 6.876 0.000 0.242 0.435

C(School)[16] 0.5572 0.051 10.853 0.000 0.457 0.658

C(School)[17] 0.8781 0.053 16.422 0.000 0.773 0.983

C(School)[18] 0.4857 0.054 9.058 0.000 0.381 0.591

C(School)[19] 0.3778 0.062 6.044 0.000 0.255 0.500

C(School)[20] 0.0045 0.069 0.066 0.948 -0.130 0.139

C(School)[21] -0.0580 0.060 -0.971 0.332 -0.175 0.059

C(School)[22] -1.3721 0.056 -24.629 0.000 -1.481 -1.263

C(School)[23] -0.1213 0.062 -1.967 0.049 -0.242 -0.000

C(IDACIQuintile, Treatment(reference="Q5"))[T.Q1] -0.4233 0.036 -11.616 0.000 -0.495 -0.352

C(IDACIQuintile, Treatment(reference="Q5"))[T.Q2] -0.3866 0.035 -11.088 0.000 -0.455 -0.318

C(IDACIQuintile, Treatment(reference="Q5"))[T.Q3] -0.3430 0.034 -10.178 0.000 -0.409 -0.277

C(IDACIQuintile, Treatment(reference="Q5"))[T.Q4] -0.2166 0.032 -6.781 0.000 -0.279 -0.154

=====================================================================================================================
